# What we can do? The risk factors for multi-drug resistant infection in pediatric intensive care unit (PICU): a case-control study

**DOI:** 10.1186/s13052-019-0769-9

**Published:** 2020-02-07

**Authors:** Zaihua Wang, Zhongfang Xia

**Affiliations:** 1 Nursing Department, Wuhan Children’s Hospital (Wuhan Maternal and Child Healthcare Hospital), Tongji Medical College, Huazhong University of Science & Technology, Wuhan, China; 2 Otolaryngology Department, Wuhan Children’s Hospital (Wuhan Maternal and Child Healthcare Hospital), Tongji Medical College, Huazhong University of Science & Technology, No 100, Xianggang Road, Wuhan, Hubei province China

**Keywords:** Risk factors, Multi-drug resistant infection, Pediatric intensive care unit

## Abstract

**Background:**

The risk factors for multi-drug resistant infection (MDRI) in the pediatric intensive care unit (PICU) remain unclear. It’s necessary to evaluate the epidemiological characteristics and risk factors for MDRI in PICU, to provide insights into the prophylaxis of MDRI clinically.

**Methods:**

Clinical data of 79 PICU children with MDRI were identified, and 80 children in PICU without MDRI in the same period were selected as control group. The related children’s characteristics, clinical care, microbiologic data, treatments provided, and outcomes of the patients with were reviewed and collected. Univariate and multivariate logistic regression analyses were performed to identify the potential risks of MDRI in PICU.

**Results:**

Of the diagnosed 79 cases of MDRI, there were28 cases of CR-AB, 24 cases of MRSA, 22 cases of PDR-PA,3 cases of VRE and 2 cases of CRE respectively. Univariate analyses indicated that the length of PICU stay, the duration of mechanical ventilation > 5 days, parenteral nutrition, coma, urinary catheter indwelling, invasive operation, 2 or more antibiotics use were associated with MDRIs (all *p* < 0.05); The logistic multiple regression analyses indicated that coma, parenteral nutrition, 2 or more antibiotics use and the duration of mechanical ventilation > 5 days were independent risk factors associated with MDRI (all *p* < 0.05).

**Conclusions:**

This present study has identified several potentially modifiable risk factors for MDRI in PICU, it’s conducive to take appropriate measures targeting risk factors of MDRI for health care providers to reduce MDRI.

## Background

In recent years, the incidence and drug resistance of multi-drug resistant infections (MDRI) have shown a rapid growth trend [1]. Particularly, the emergence of a large number of pan-drug resistant strains poses great difficulties for treatment of pediatric patients [2]. The intensive care unit has become a high-risk area for hospital-acquired infections and drug-resistant strains regarding its large number of special susceptible populations and its special diagnosis and treatment environment, especially in the pediatric intensive care unit (PICU) [3]. PICU patients are particularly susceptible to nosocomial infections, the potential reasons may include that the use of invasive devices and procedures in this critically ill patient group, and the children’s immune function is relatively incompletely developed [4]. Although a serious infection may prompt the entry to PICU, MDRI may be a post-hospital complication, potentially life threatening for children [5]. Many previous studies [6–8] have indicated that the incidence of nosocomial infections caused by antibiotic-resistant organisms is increasing. The prevalence of hospital-acquired or nosocomial infections in pediatric patients ranges from 10 to 25% in PICU [9, 10]. Therefore, the control of infection in PICU is very important.

Understanding the reasons behind that is very essential. Certain risk factors are associated with nosocomial infections caused by drug-resistant organisms. In adult patients with interstitial transfer, long-term hospitalization, gastrointestinal surgery and transplantation, as well as exposure to all types of invasive devices and exposure to previous antimicrobial agents are closely associated with MDRI in ICU [11]. It’s been reported [12] that *Staphylococcus aureus* (MRSA), vancomycin-resistant Enterococcus (VRE), Carbapenem-based antibacterial drug Acinetobacter baumannii (CR-AB) are the three commonly-seen multi-drug resistant bacteria. In PICU, considering that the low immunity, serious condition, long hospitalization, mechanical ventilation, invasive examination and other factors related to the treatment of children, the incidence of MDRIs increases significantly, posing a great threat to children [4, 13].

Therefore, it is necessary to analyze the risk factors for MDRIs in PICU, to understand characteristics of MDRIs in PICU, thereby providing a basis for developing related treatment and nursing strategies for the prophylaxis and management of MDRIs in PICU.

## Methods

### Study design

A cases and controls study.

### Setting

Three PICUs of one tertiary children’ hospital were included, of which the microbial identification results are homogeneous and are regulatory accepted by each other. Nine hundred six patients who were hospitalized in ICU during the period of July 1st 2018 to June 30th 2019 were identified, of which 79 Children were diagnosed as MDRI. Therefore, we included those 79 children with MDRI as participants in the present study, and repeated strains isolated from the same patient in the same part of the specimen were excluded. In the same period, 80 children in PICU without MDRI were selected as the control group, we have included the children with the same diagnosis with the cases as controls.

We used following methods to control the quality of sputum samples: when being under laboratory microscope, the squamous epithelial cells in the low fold field of vision < 10 and the white blood cells > 25 were defined as qualified samples. And all the specimens were sent to the laboratory room for bacteria analysis.

### Definitions

MDRI was defined as having an isolate resistant to more than three kinds of the following antimicrobial or antimicrobial groups: ampicillin/sulbactam, aztreonam, ceftazidime, ciprofloxacin, gentamicin, piperacillin, trimethoprim/sulfamethoxazole, carbapenem and amikacin [14, 15]. Bacterial isolation and antimicrobial susceptibility testing were performed according to the method of the Clinical and Laboratory Standards Institute [16].

Invasive operation was defined as having inserted or indwelling catheters or tubes, such as the status of mechanic ventilation.

Drug sensitivities was conditions which exist due to the natural variation in patients’ metabolism of drugs. Some people may react to them faster than others, and some people may have a lower threshold to the effects of drugs than others. The effects caused by this lower threshold was defined as drug sensitivities, which was performed in the laboratory room in our hospital.

### Data collection

The cases were reviewed in the microbiology and inspection laboratory database by searching for at least 2 positive cultures. And the medical records of the cases were obtained from the medical record archives. The demographics-related information, regarding clinical care, microbiologic data, treatments provided, and outcomes of the patients with were reviewed and collected. Main contents including the name, gender, age, the length of ICU stays, infection site, operation, invasive operation, antibiotic use, and drug sensitivity were collected. All the data were analyzed after double-entry check.

### Statistical analysis

All of the statistical analyses were conducted using SPSS 21.0 (SPSS Inc., Chicago, USA). Categorical variables were analyzed using the χ^2^ test or Fisher’s exact test, and continuous variables were analyzed using Student’s t test or Mann-Whitney U test, and were generally presented as means and standard deviation. Multivariate logistic regression analyses were performed using the forward likelihood ratio selection method to identify independent factors of MDRI and it is presented with an odds ratio (95% confidence intervals, CI). Potential candidate variables were those with *P* < 0.05 in univariate analyses. All of the *P* values were 2 tailed, and P < 0.05 was considered as being statistically significant.

## Results

### The distribution of MDRI bacteria

**Fig. 1:**
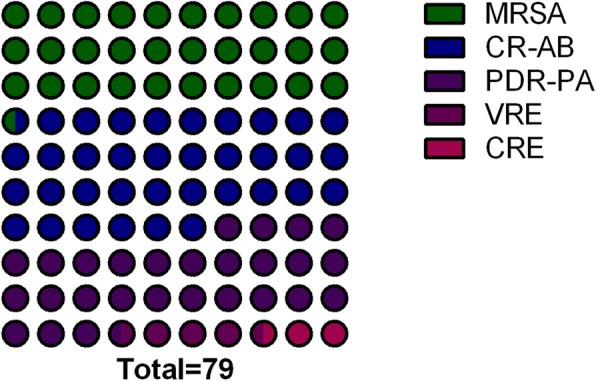
The distribution of infected multidrug-resistant bacteria

Of the diagnosed 79 cases of MDRI, there were 28 cases of CR-AB, 24 cases of MRSA, 22 cases of PDR-PA,3 cases of VRE and 2 cases of CRE respectively. The distribution of infected multidrug-resistant bacteria is presented in Fig. 1.

### The source of MDRIs from clinical specimens

As Table 1 showed, of the 79 cases of MDRIs, 43 cases were detected in the whole blood, 23 cases in sputum, 5 cases in drainage fluid, 5 cases in central venous catheter tip and 3 cases in other source.

**Table 1:** Source of MDRIs from clinical specimens

Bacteria	Whole blood	Sputum	Drainage fluid	Central venous catheter tip	Others
CR-AB(*n* = 28)	19	5	2	2	0
MRSA(*n* = 24)	4	15	1	3	1
PDR-PA(*n* = 22)	17	2	1	0	2
VRE(*n* = 3)	2	0	1	0	0
CRE(*n* = 2)	1	1	0	0	0
Total	43	23	5	5	3

### Univariate analysis on the risk factors of MDRIs

As Table 2 presented, The length of PICU stay, the duration of mechanical ventilation > 5 d, parenteral nutrition, coma, urinary catheter indwelling, invasive operation, 2 or more antibiotics use were associated with MDRIs (all *p* < 0.05), but the gender, age, mechanical ventilation, PICC insertion, application of hormones were not associated with MDRIs (all *p* > 0.05).

**Table 2:** Univariate analysis on the risk factors for the infection of multidrug-resistant bacteria

Factors	Infection group (*n* = 74)	Control group (*n* = 80)	χ^2^/t	*p*
Male/female	41/33	44/36	13.07	0.052
Age (years)	5.08 ± 1.37	5.12 ± 1.18	2.40	0.065
The length of PICU stay (days)	30.48 ± 5.11	15.09 ± 3.85	18.49	0.007
Mechanical Ventilation	46	52	4.29	0.053
The duration of mechanical ventilation > 5 d	30	2	6.03	0.001
PICC insertion	39	36	4.11	0.071
Parenteral nutrition	63	10	11.04	0.010
Coma	28	11	8.25	0.016
Urinary catheter indwelling	40	17	4.39	0.008
Application of hormones	49	42	15.30	0.084
Invasive operation	68	27	8.49	0.012
2 or more antibiotics use	52	25	10.67	0.009

### Multivariate regression analysis on the risk factors of MDRIs

Unconditional logistic multiple regression analyses were further performed on the seven variables with significant differences in univariate analysis, the logistic multiple regression analyses indicated that coma, parenteral nutrition, 2 or more antibiotics use and the duration of mechanical ventilation > 5 d were independent risk factors associated with MDRIs respectively (Table 3).

**Table 3:** Logistic regression analysis on the factors for the infection of multidrug-resistant bacteria

Factors	β	S^−^x	OR	95%CI	p	Rank
Coma	0.89	0.29	4.38	1.15~9.53	0.048	1
Parenteral nutrition	0.92	0.31	5.31	1.23~10.75	0.031	2
2 or more antibiotics use	1.03	0.45	4.97	1.37~9.86	0.042	3
The duration of mechanical ventilation > 5 d	0.99	0.39	5.67	2.24~13.42	0.019	4

### Subgroups sensitivity analyses

We didn’t perform any subgroup analyses with consideration to that the sample size in this present study is very limited. However, we did sensitivity analyses by removing the related data of included participant one by one to detect the potential results bias, and no significantly different results were found.

## Discussion

In this present study, the incidence of MDRIs in the three PICUs was 8.72% (79/906), which is significantly lower than that of previous reports with incidence of MDRIs ranging from 10 to 25% in PICU [9, 10]. Previous studies [17–19] have reported that the patients with MDRI were hospitalized and treated in the ICU for considerably longer stay and had lower survival rates compared to other patient groups. Identifying risk factors for infection development caused by multidrug-resistant bacteria can help health care providers prevent nosocomial infections. This becomes even more important if we consider the slow drug development of new effective anti-bacteria and the rising prevalence of MDRI, especially in the PICU. The results of this present study have revealed that coma, parenteral nutrition, 2 or more antibiotics use and the duration of mechanical ventilation > 5 d are independent risk factors associated with MDRIs in PICU. To the best of our knowledge, very few studies focus on the MDRIs in PICU, so we believe that the present study can provide some evidences for the prophylaxis of MDRIs.

Infected patients are mainly concentrated in the coma population of children. Coma patients have higher risk of infection due to more basic diseases and low immunity [20]. There are many types of diseases in the ICU, the coma children in PICU are usually seriously ill and the treatment periods are longer [2, 21]. The extension of the ICU stay increases the use of antibiotics, invasive operation and cross-infection between patients. Besides, the body’s own pathogens may shift to other tissues and organs, leading to endogenous infections [22]. As the hospitalization period is extended, the patient’s medical expenses will also increase, and the human-borne pathogens can invade the body, causing exogenous infections, thus the risk of MDRI can increase [23]. Usually, the beds in the ICU are very few, and the increase of MDRI makes the medical resources not properly configured. Previous studies [24, 25] have concluded that the hospitalization time of patient needs to be strictly controlled, which is one of the effective measures to prevent MDRI.

Mechanical ventilation can significantly increase the chance of respiratory infection [26, 27]. The establishment of an artificial airway in an ICU patient results in the disappearance of the natural barrier of the upper respiratory tract and direct communication of the lower respiratory tract with the outside air. Various invasive medical procedures potentially increase the risk of infection in the lower respiratory tract [28]. The establishment of the artificial airway opens the respiratory pathway for the patient, but long-term mechanical ventilation and open-ended invasive procedures such as suction and fiberoptic bronchoscopy increase the incidence of VAP [29]. Additionally, resistant bacteria can easily enter the bloodstream through the damaged blood gas barrier, causing blood sepsis [30]. Besides, it is often the case that the maintenance and care of the ventilator tube is not standardized, or the equipment is not properly disinfected, which provides a shortcut for the invasion of the drug-resistant bacteria, which is one of the important reasons for the MDRI in ICU [31, 32]. In clinical setting, special attention should be paid to the patient in comatose state and with mechanical ventilation length > 5 days. Patients who have been in bed for a long time are prone to pneumonia.

The use of antibacterial drugs is a key factor related to bacterial resistance [33, 34]. The selective pressure of bacteria comes from the use of antibiotic dose [35]. When the antibiotic used by the patient reaches or is about to reach the sublethal dose, the selective pressure of the antibiotic can force the change of body’s behavior, physiology and biochemistry [36]. The gene and protein expression of the resistant bacteria will change, the protective defense and immunity of the body will gradually weaken, and the risk of MDRI elevates [37]. China is a large country with massive use of antibiotics, and the frequency of antibiotics use is very high [38, 39]. It’s been reported [40, 41] that almost 80% hospitalized patients in China have been prescribed antibacterial drugs, and the use of broad-spectrum antibiotics is 28% higher than the average antibiotics use globally. There is a close correlation between the amount of antibiotics used and bacterial resistance. ICU has higher pathogen composition ratio and bacterial resistance [34], thus it’s necessary to fully grasp the basic information of bacterial epidemiology in ICU, and control the resistance of various drug-resistant bacteria with different antibiotics, to provide basis for MDRI treatment and strategies.

It must be mentioned that the findings of our study should be interpreted with the consideration of potential limitations. Firstly, it should be acknowledged that, due to the innate limitations of retrospective studies, a significant proportion of the children had more than one incidence of infection, but only one incidence per patient was analyzed. Secondly, there is ample room for improvement in the explanation of the rationale to include cases, and specially, for the justification of choosing a case: control ratio of 1:1, when the customary ratio is to have at least 2 controls for each case. The relatively limited number of cases included in this present analysis may not be powered enough to detect the potential risk factors for MDRIs, future studies with larger sample and broader areas are needed to identify the MDRIs risks. Despite these shortcomings in this study, the results of this study may still lend some inspiration for clinical health workers, as this is the first analysis of the risk factors for the development MDRI in PICU.

In conclusion, this present study has revealed that coma, parenteral nutrition, 2 or more antibiotics use and the duration of mechanical ventilation > 5 days are independent risk factors associated with MDRIs in PICU. Based on the evidence provided in this study, specific actions can be taken to improve prevention of MDRIs, such implementing the post of infection control nurses et al. [42], which may significantly reduce the MDRIs. The evidences provided can help critical care providers in PICU determine the extent to which they can modify their treatment strategies to achieve optimal clinical outcomes.

## Data Availability

All data generated or analyzed during this study are included in this published article.

## Abbreviations

MDRI: Multi-drug resistant infections
PICU: Pediatric intensive care unit

## References

[1] Liu C, Yoon EJ, Kim D, Shin JH, Shin JH, Shin KS, Kim YA, Uh Y, Kim HS, Kim YR (2019). Antimicrobial resistance in South Korea: A report from the Korean global antimicrobial resistance surveillance system (Kor-GLASS) for 2017. J Infect Chemother.

[2] Atay G, Kara M, Sutcu M, Aydin YS, Torun SH, Karapinar BA, Kayacan ZC, Gurler N, Citak A, Nisli K (2019). Resistant gram-negative infections in a pediatric intensive care unit: a retrospective study in a tertiary care center. Turk Pediatri Ars.

[3] Ganesh R, Shrestha D, Bhattachan B, Rai G (2019). Epidemiology of urinary tract infection and antimicrobial resistance in a pediatric hospital in Nepal. BMC Infect Dis.

[4] Aygun, Aygun, Varol, Durak, Cokugraş, Camcioglu, Cam (2019). Can Nebulised Colistin Therapy Improve Outcomes in Critically Ill Children with Multi-Drug Resistant Gram-Negative Bacterial Pneumonia?. Antibiotics.

[5] Fu Q, Ye H, Liu S (2015). Risk factors for extensive drug-resistance and mortality in geriatric inpatients with bacteremia caused by Acinetobacter baumannii. Am J Infect Control.

[6] Chaisathaphol T, Chayakulkeeree M (2014). Epidemiology of infections caused by multidrug-resistant gram-negative bacteria in adult hospitalized patients at Siriraj hospital. J Med Assoc Thail.

[7] Medell M, Hart M, Duquesne A, Espinosa F, Valdes R (2013). Nosocomial ventilator-associated pneumonia in Cuban intensive care units: bacterial species and antibiotic resistance. MEDICC Rev.

[8] Falagas ME, Bliziotis IA, Kasiakou SK, Samonis G, Athanassopoulou P, Michalopoulos A (2005). Outcome of infections due to pandrug-resistant (PDR) gram-negative bacteria. BMC Infect Dis.

[9] Peng H, Wang X, Yan X, Zhao X (2015). Risk factors analysis and nursing countermeasures of multiple drug resistant bacteria infection in PICU. Chin Nurs Res.

[10] Zheng B, Dai Y, Liu Y, Shi W, Dai E, Han Y, Zheng D, Yu Y, Li M (2017). Molecular epidemiology and risk factors of Carbapenem-resistant Klebsiella pneumoniae infections in eastern China. Front Microbiol.

[11] Cucci M, Wooten C, Fowler M, Mallat A, Hieb N, Mullen C. Incidence and risk factors associated with multi-drug-resistant pathogens in a critically ill trauma population: a retrospective cohort study. Surg Infect. 2019;12:11–6. 10.1089/sur.2019.031 31210580

[12] Reale M, Strazzulla A, Quirino A, Rizzo C, Marano V, Postorino MC, Mazzitelli M, Greco G, Pisani V, Costa C (2017). Patterns of multi-drug resistant bacteria at first culture from patients admitted to a third level university hospital in Calabria from 2011 to 2014: implications for empirical therapy and infection control. Infez Med.

[13] El-Nawawy A, Ramadan MA, Antonios MA, Arafa SA, Hamza E (2019). Bacteriologic profile and susceptibility pattern of mechanically ventilated paediatric patients with pneumonia. J Glob Antimicrob Resist.

[14] Zarrilli R, Pournaras S, Giannouli M, Tsakris A (2013). Global evolution of multidrug-resistant Acinetobacter baumannii clonal lineages. Int J Antimicrob Agents.

[15] Taitt CR, Leski TA, Stockelman MG, Craft DW, Zurawski DV, Kirkup BC, Vora GJ (2014). Antimicrobial resistance determinants in Acinetobacter baumannii isolates taken from military treatment facilities. Antimicrob Agents Chemother.

[16] Jorgensen JH, Hindler JF (2007). New consensus guidelines from the clinical and laboratory standards institute for antimicrobial susceptibility testing of infrequently isolated or fastidious bacteria. Clin Infect Dis.

[17] McGrath EJ, Asmar BI (2011). Nosocomial infections and multidrug-resistant bacterial organisms in the pediatric intensive care unit. Indian J Pediatr.

[18] Jeena P, Thompson E, Nchabeleng M, Sturm A (2001). Emergence of multi-drug-resistant Acinetobacter anitratus species in neonatal and paediatric intensive care units in a developing country: concern about antimicrobial policies. Ann Trop Paediatr.

[19] Hu J, Robinson JL (2010). Systematic review of invasive Acinetobacter infections in children. Can J Infect Dis Med Microbiol.

[20] Ono Y, Ono S, Yasunaga H, Matsui H, Fushimi K, Tanaka Y (2017). Clinical characteristics and outcomes of myxedema coma: analysis of a national inpatient database in Japan. J Epidemiol.

[21] Kuo CC, Lee YS, Lin MR, Hsia SH, Chen CJ, Chiu CH, Hwang MS, Huang YC (2018). Characteristics of children with Kawasaki disease requiring intensive care: 10 years’ experience at a tertiary pediatric hospital. J Microbiol Immunol Infect.

[22] Zhang XX, Geng ZX, Zhu L, Li MH, Wang YJ, Qian SY (2019). Liu G: [clinical analysis of children with group B streptococcal meningitis in 2013-2017 in a single center]. Zhonghua Er Ke Za Zhi.

[23] Nadeau N, Monuteaux MC, Tripathi J, Stack AM, Perron C, Neuman MI (2019). Pediatric ICU transfers within 24 hours of admission from the emergency department: rate of transfer, outcomes, and clinical characteristics. Hosp Pediatr.

[24] Reinheimer C, Kempf VA, Jozsa K, Wichelhaus TA, Hogardt M, O'Rourke F, Brandt C (2017). Prevalence of multidrug-resistant organisms in refugee patients, medical tourists and domestic patients admitted to a German university hospital. BMC Infect Dis.

[25] Ma X, Wu Y, Li L, Xu Q, Hu B, Ni Y, Wu A, Sun S, Jarlier V, Robert J (2015). First multicenter study on multidrug resistant bacteria carriage in Chinese ICUs. BMC Infect Dis.

[26] Tamma PD, Turnbull AE, Milstone AM, Lehmann CU, Sydnor ER, Cosgrove SE (2011). Ventilator-associated tracheitis in children: does antibiotic duration matter?. Clin Infect Dis.

[27] Li Y, Liu C, Xiao W, Song T, Wang S. Incidence, risk factors, and outcomes of ventilator-associated pneumonia in traumatic brain injury: a meta-analysis. Neurocrit Care. 2019;24:1–5. 10.1007/s12028-019-00773-w PMC7223912 31300956

[28] Wang C, Ye S, Wang X, Zhao Y, Ma Q, Wang L (2019). Clinical efficacy and safety of mechanical ventilation combined with Fiberoptic Bronchoalveolar lavage in patients with severe pulmonary infection. Med Sci Monit.

[29] Tobin M, Manthous C (2017). Mechanical ventilation. Am J Respir Crit Care Med.

[30] Andersson DI, Hughes D. Selection and Transmission of Antibiotic-Resistant Bacteria. Microbiol Spectr. 2017;5(4):2–4. 10.1128/microbiolspec.mtbp-0013-2016 PMC11687535 28752817

[31] Peterson E, Kaur P (2018). Antibiotic resistance mechanisms in Bacteria: relationships between resistance determinants of antibiotic producers, environmental Bacteria, and clinical pathogens. Front Microbiol.

[32] Huang H, Chen B, Liu G, Ran J, Lian X, Huang X, Wang N, Huang Z (2018). A multi-center study on the risk factors of infection caused by multi-drug resistant Acinetobacter baumannii. BMC Infect Dis.

[33] Medina E, Pieper DH (2016). Tackling threats and future problems of multidrug-resistant Bacteria. Curr Top Microbiol Immunol.

[34] Sanchez-Ramirez C, Hipola-Escalada S, Cabrera-Santana M, Hernandez-Viera MA, Caipe-Balcazar L, Saavedra P, Artiles-Campelo F, Sangil-Monroy N, Lubbe-Vazquez CF, Ruiz-Santana S (2018). Long-term use of selective digestive decontamination in an ICU highly endemic for bacterial resistance. Crit Care.

[35] Penchovsky R, Traykovska M (2015). Designing drugs that overcome antibacterial resistance: where do we stand and what should we do?. Expert Opin Drug Discov.

[36] Ciofi Degli Atti ML, D’Amore C, Ceradini J, Paolini V, Ciliento G, Chessa G, Raponi M (2019). Prevalence of antibiotic use in a tertiary care hospital in Italy, 2008-2016. Ital J Pediatr.

[37] Ji Y, Lei T (2013). Antisense RNA regulation and application in the development of novel antibiotics to combat multidrug resistant bacteria. Sci Prog.

[38] Cui D, Liu X, Hawkey P, Li H, Wang Q, Mao Z, Sun J (2017). Use of and microbial resistance to antibiotics in China: a path to reducing antimicrobial resistance. J Int Med Res.

[39] Bu Q, Wang B, Huang J, Liu K, Deng S, Wang Y, Yu G (2016). Estimating the use of antibiotics for humans across China. Chemosphere.

[40] Zhen, Lundborg, Sun, Hu, Dong (2019). The Clinical and Economic Impact of Antibiotic Resistance in China: A Systematic Review and Meta-Analysis. Antibiotics.

[41] He P, Sun Q, Shi L, Meng Q (2019). Rational use of antibiotics in the context of China's health system reform. BMJ.

[42] Xu W, He L, Liu C, Rong J, Shi Y, Song W, Zhang T, Wang L (2015). The effect of infection control nurses on the occurrence of Pseudomonas aeruginosa healthcare-acquired infection and multidrug-resistant strains in critically-ill children. PLoS One.

